# PD-1 defines a distinct, functional, tissue-adapted state in Vδ1^+^ T cells with implications for cancer immunotherapy

**DOI:** 10.1038/s43018-023-00690-0

**Published:** 2024-01-03

**Authors:** Daniel Davies, Shraddha Kamdar, Richard Woolf, Iva Zlatareva, Maria Luisa Iannitto, Cienne Morton, Yasmin Haque, Hannah Martin, Dhruva Biswas, Susan Ndagire, Martina Munonyara, Cheryl Gillett, Olga O’Neill, Oliver Nussbaumer, Adrian Hayday, Yin Wu

**Affiliations:** 1 Peter Gorer Department of Immunobiology, King’s College London, London, UK; 2 Centre for Inflammation Biology and Cancer Immunology, King’s College London, London, UK; 3 St. John’s Institute of Dermatology, Guy’s Hospital, London, UK; 4 Department of Medical Oncology, Guy’s Hospital, London, UK; 5 Immunosurveillance Laboratory, Francis Crick Institute, London, UK; 6 Academic Foundation Programme, King’s College Hospital, London, UK; 7 King’s Health Partners Cancer Biobank, Guy’s Hospital, London, UK; 8 Department of Cellular Pathology, St. Thomas’ Hospital, London, UK; 9 Advanced Sequencing Facility, Francis Crick Institute, London, UK

**Keywords:** Cancer immunotherapy, Tumour immunology, Cancer

## Abstract

Checkpoint inhibition (CPI), particularly that targeting the inhibitory coreceptor programmed cell death protein 1 (PD-1), has transformed oncology. Although CPI can derepress cancer (neo)antigen-specific αβ T cells that ordinarily show PD-1-dependent exhaustion, it can also be efficacious against cancers evading αβ T cell recognition. In such settings, γδ T cells have been implicated, but the functional relevance of PD-1 expression by these cells is unclear. Here we demonstrate that intratumoral *TRDV1* transcripts (encoding the TCRδ chain of Vδ1^+^ γδ T cells) predict anti-PD-1 CPI response in patients with melanoma, particularly those harboring below average neoantigens. Moreover, using a protocol yielding substantial numbers of tissue-derived Vδ1^+^ cells, we show that PD-1^+^Vδ1^+^ cells display a transcriptomic program similar to, but distinct from, the canonical exhaustion program of colocated PD-1^+^CD8^+^ αβ T cells. In particular, PD-1^+^Vδ1^+^ cells retained effector responses to TCR signaling that were inhibitable by PD-1 engagement and derepressed by CPI.

## Abstract (web summary)

Davies et al. show that skin PD-1^+^Vδ1^+^ cells show a transcriptional program of tissue residence and self-renewal, are functionally distinct from colocalized PD-1^+^CD8^+^ T cells and retain effector function that is derepressed by checkpoint blockade.

## Main

Checkpoint inhibition (CPI) therapies have transformed how advanced cancers are managed, offering many patients the prospect of durable remission and even cure^1,2^. These therapies are widely thought to act by derepressing dysfunctional or exhausted (neo)antigen-specific αβ T cells. Among myriad CPI modalities, those blocking interactions of programmed cell death protein 1 (PD-1) with its ligand, programmed death ligand 1 (PD-L1), have consistently been the most effective^3,4^. Nonetheless, anti-PD-1 and anti-PD-L1 CPI therapies benefit only a minority of patients. To improve these response rates, much effort has been devoted to a better understanding of the biology of PD-1 in the context of αβ T cells and particularly cytotoxic CD8^+^ αβ T cells (hereafter referred to as CD8^+^ T cells) because those cells have been implicated in favorable responses to anti-PD-1 and anti-PD-L1 therapies^5,6^. While PD-1 has historically been viewed as an inhibitory coreceptor expressed by chronically activated CD8^+^ T cells differentiating toward terminal exhaustion, recent studies suggest it is also important for the survival of CD8^+^ T cells within tissues and for the maintenance of a stem-like, ‘pre-exhausted’ progenitor population^7–9^. However, for reasons considered below, attention has recently been devoted to the prospect that substantial clinical benefits of anti-PD-1 and anti-PD-L1 CPI therapies may derive from their impact on other T cells, particularly γδ T cells.

Like CD8^+^ T cells, γδ T cells include cytotoxic T cells that can express PD-1 (refs. ^10–14^). However, unlike CD8^+^ T cells, γδ T cells can detect cancer cells by engaging the T cell (TCR) or natural killer (NK) receptors without any obligate requirement for cognate cancer neoantigens presented on self-major histocompatibility complex (MHC)^15^. This is important, because many cancers have low mutational loads with a corresponding paucity of neoantigens and many have defects in MHC antigen presentation^16–21^. Hence, whereas these cancers are de facto concealed from CD8^+^ T cells, they may be visible to γδ T cells, possibly explaining the fact that intratumoral γδ T cells, particularly tissue-associated Vδ1^+^ cells, have consistently been linked to favorable clinical outcomes, for example, improved survival, in many cancer settings often independent of colocated αβ T cells^10,11,22–24^.

More recently, several studies have implicated Vδ1^+^ cells as putative effectors in clinical anti-PD-1 and anti-PD-L1 therapy^13,14,23^. We and others have shown that the intratumoral transcriptomic presence of these cells associates with response to anti-PD-1 and anti-PD-L1 CPI therapies in several cancer types^14,23^. Moreover, Vδ1^+^ cell-associated transcripts in colorectal tumors have been shown to significantly increase after neoadjuvant anti-PD-1 CPI therapy^13^. However, while these findings are provocative and potentially profound, they exist in a context lacking evidence for any immunological relevance of PD-1 expression by Vδ1^+^ cells. Critically, whether Vδ1^+^ cells can be inhibited by PD-1 engagement and if so, whether anti-PD-1 and anti-PD-L1 CPI therapies can derepress this are unknown. Indeed, the commonly perceived utility of γδ T cells for immunotherapy is their capacity to kill transformed cells independent of their TCR^15,24^, yet PD-1 attenuation of αβ T cell function is classically downstream of TCR signaling^25^. In part, the unresolved immunological relevance of PD-1 expression by Vδ1^+^ cells reflects the challenges of isolating the cells from primary human tissues in sufficient numbers for study. In this study, we overcome those challenges, thereby combining direct experimentation with correlative clinical evidence to show that PD-1^+^ Vδ1^+^ cells can be a target effector population of anti-PD-1 and anti-PD-L1 CPI therapy. Among other things, these findings offer a clear rationale for adding anti-PD-1 and anti-PD-L1 CPI to clinical trials of ongoing Vδ1^+^ adoptive cell therapies (ACTs).

## Results

### Transcriptomic presence of Vδ1^+^ cells predicts CPI response

**Extended Data Fig. 1:**
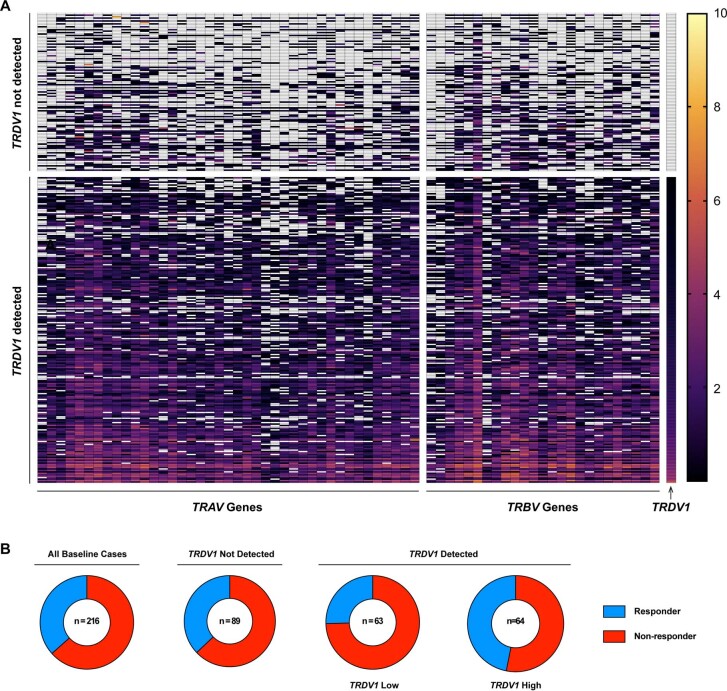
RNAseq TCR V gene alignment. **A**, Heatmap of *TRAV* and *TRBV* genes detected across all five melanoma public datasets analysed for Fig. 1 segregated by *TRDV1* status (detected versus not detected) demonstrating a coordinated global lack of TCR V gene alignment. Unfilled cells represent undetected genes. Colour scale denotes Log2(TPM + 1) gene expression. **B**, Objective response rates of entire cohort of baseline cases (n = 216), baseline cases where *TRDV1* was not detected (n = 89) and cases where *TRDV1* was either below (*TRDV1* low, n = 63) or above (*TRDV1* high, n = 64) median (see Methods). Source data

Melanoma is the archetypal cancer in which CPI therapy has been developed and applied^26^, but in which there remains a challenge of broadening its efficacy. Moreover, the skin is the archetypal tissue in which human Vδ1^+^ cells have been studied^27–29^. Thus, we first sought to build an evidence-base for whether Vδ1^+^ cells might have a role in anti-PD-1 and anti-PD-L1 CPI therapy in melanoma. To this end, we evaluated public transcriptomic data from patients with advanced melanoma treated with anti-PD-1 or anti-PD-L1. We identified five studies with publicly accessible aligned RNA sequencing (RNA-seq) data and matched clinical response criteria^30–34^ from a recent review^35^ and meta-analysis of CPI transcriptomic datasets^34^ yielding a total of 216 suitable cases (Methods). Next, we assessed tumors for their expression of the *TRDV1* gene, which is a widely accepted robust surrogate for Vδ1^+^ cells^10,11,13,36^ because it encodes the Vδ1 TCR subunit and is commonly deleted in αβ T cells^37^. Approximately 40% of tumors (89 of 216; Methods) had no detectable *TRDV1* expression and this was conspicuously associated with a deficit of detectable TCRα and TCRβ V genes (*TRAV* and *TRBV*; Extended Data Fig. 1a), probably reflecting immune-cold, that is, T cell-excluded tumors. Consistent with this, patients with tumors in which *TRDV1* could not be detected had the same response rate to anti-PD-1 and anti-PD-L1 CPI therapy as the unselected cohort, whereas cases with detectable *TRDV1* showed a clear segregation of response with the degree of *TRDV1* expression (Extended Data Fig. 1b), justifying further outcome analyses based on cases in which *TRDV1* could be detected.

**Fig. 1:**
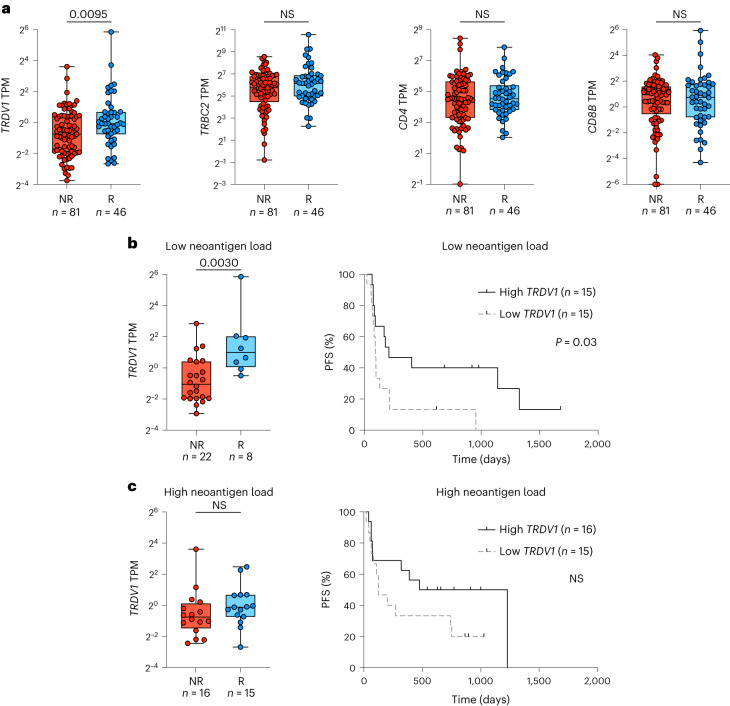
Transcriptomic presence of Vδ1^+^ cells within melanoma predicts the response to anti-PD-1 and anti-PD-L1 CPI therapy. **a**, Expression (transcripts per million (TPM)) of *TRDV1*, *TRBC2*, *CD4* and *CD8B* plotted according to the objective response to anti-PD-1 and anti-PD-L1 CPI therapy. Mann–Whitney *U*-test. **b**, Left, expression of *TRDV1* in tumors with below-median neoantigen loads from Liu et al.^33^ plotted according to the objective response to anti-PD-1 CPI therapy. Right, progression-free survival (PFS) of patients with below-median neoantigen loads from Liu et al.^33^ split on the median expression of *TRDV1*. log-rank test. **c**, Left, expression of *TRDV1* in tumors with above-median neoantigen loads from Liu et al.^33^ plotted according to the objective response to anti-PD-1 CPI therapy. Mann–Whitney *U*-test. Right, PFS of patients with above-median neoantigen loads from Liu et al.^33^ split on the median expression of *TRDV1*. log-rank test. All *P* values presented are two-sided where relevant. For the box plots, the boxes denote the medians and interquartile ranges (IQRs); the whiskers denote the minimum and maximum values. NR, nonresponder; NS, not significant; R, responder. Source data

Of these cases (*n* = 127), we found that tumors from patients with response to treatment expressed significantly higher levels of *TRDV1* than tumors from patients without response. Conversely, this was not observed for *TRBC2*, *CD4* or *CD8B*, which are broadly accepted surrogates for CD4^+^ and CD8^+^ αβ T cells (Fig. 1a). Given the capacity of Vδ1^+^ cells to act independently of TCR neoantigen engagement^10,15,24^, we further analyzed the association of *TRDV1* with CPI response stratified according to neoantigen load in the largest public dataset^33^ (Methods). Strikingly, the correlation of high *TRDV1* expression with benefit from anti-PD-1 CPI therapy was most evident in tumors with below-median neoantigen loads (Fig. 1b,c). Together, these observations supported the hypothesis that Vδ1^+^ cells can be derepressed by anti-PD-1 and anti-PD-L1 CPI therapy, thus providing a protective role for patients with cancer. As a critical test of this hypothesis, we sought direct evidence for a functional role of PD-1 on human tissue-derived Vδ1^+^ cells.

### PD-1^+^Vδ1^+^ cells are distinct from their CD8^+^ counterparts

**Fig. 2:**
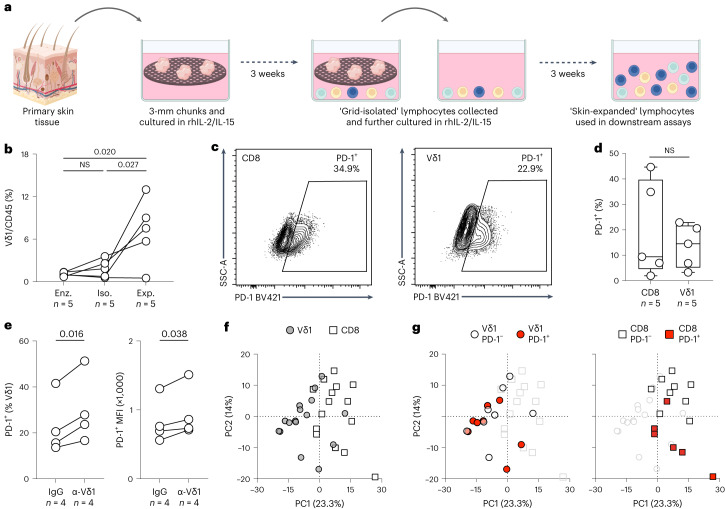
Expression of PD-1 on Vδ1^+^ cells is associated with distinct biology compared with its expression on CD8^+^ T cells. **a**, Schematic of nonenzymatic extraction of lymphocytes from primary human tissue and subsequent expansion protocol. **b**, Summary flow cytometry data of Vδ1^+^ cell enrichment in skin-expanded (Exp.) lymphocytes compared with skin lymphocytes obtained by direct enzymatic digestion (Enz.) and grid isolation (Iso.). Repeated measures one-way analysis of variance (ANOVA) followed by Holm–Šídák’s multiple comparisons test. Adjusted *P* values as indicated. **c**, Representative flow cytometry plot of PD-1 expression on skin-expanded CD8^+^ T cells (left) and Vδ1^+^ cells (right). PD-1 gates were set on paired unstained samples. Representative of *n* = 5 independent donors. **d**, Summary flow cytometry data of PD-1 expression on Vδ1^+^ and CD8^+^ T cells in skin-expanded lymphocytes from *n* = 5 independent donors. Paired *t*-test. The boxes denote the medians and IQRs and the whiskers denote the minimum and maximum values. **e**, Summary flow cytometry data of PD-1 expression on skin-expanded Vδ1^+^ cells from *n* = 4 independent donors after 48-h culture in vitro in the presence of plate-bound IgG (control) or anti-TCR-Vδ1 antibody. Results were plotted as the percentage of Vδ1^+^ cells positive for PD-1 (left) and mean fluorescence intensity (MFI) of the PD-1^+^ Vδ1 population (right). Data points are paired according to donor. Paired *t*-test. **f**, PCA of cell populations sorted from skin-expanded lymphocytes from healthy skin of independent donors based on normalized counts of all 757 genes in the nCounter Immune Exhaustion Panel (excluding γδ and CD8^+^ T cell lineage genes and *PDCD1*; Methods) highlighted according to cell type (Vδ1^+^ cells, gray circles; CD8^+^ T cells, white squares). **g**, PCA of cell populations sorted from skin-expanded lymphocytes highlighted according to PD-1 status. Left, PD-1^−^Vδ1^+^ (white circles, *n* = 8 independent donors) and PD-1^+^Vδ1^+^ (red circles, *n* = 8 independent donors) cells are highlighted. Right, PD-1^−^CD8^+^ T (white squares, *n* = 9 independent donors) and PD-1^+^CD8^+^ T (red squares, *n* = 6 independent donors) cells are highlighted. All *P* values presented are two-sided. Source data

**Extended Data Fig. 2:**
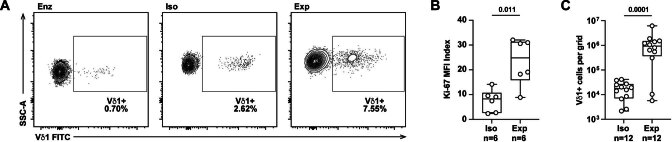
Grid isolation and expansion enriches for skin-derived Vδ1+ cells and improves yield. **A**, Representative flow cytometry dot plots demonstrating Vd1+ cell enrichment in skin-expanded lymphocytes (Exp) compared with direct enzymatic digestion (Enz) and grid isolation (Iso). Gated on live, single, CD45+ cells. Representative of n = 5 independent donors. **B**, Summary flow cytometry data of intracellular Ki-67 staining in grid-isolated Vδ1+ cells (Iso) and after 14 days of expansion (Exp). Ki-67 MFI index calculated as Ki-67 MFI for the whole Vδ1+ population divided by the MFI of the isotype control for the same population. Paired t test. **C**, Summary data of absolute numbers of Vδ1+ cells obtained from a single grid at the end of 3 weeks of grid isolation (Iso) and at the end of 3 weeks of expansion (Exp). Ratio paired t test. Datapoints represent individual skin donors. All P values presented are two-sided. For box plots, boxes denote medians and interquartile ranges, and whiskers denote minimum and maximum values. Source data

To achieve this, we needed to isolate sufficient human Vδ1^+^ cells for further studies. Because these cells are relatively rare, we adapted a nonenzymatic in vitro explant system previously demonstrated to facilitate the extraction and characterization of large numbers of human tissue-resident αβ T cells^38^ and tissue-resident γδ T cells with phenotypes comparable to direct, low-yield enzymatic disaggregation protocols^10^ (Fig. 2a). We applied this method to human skin samples residual to surgical reconstructions or abdominoplasties. Samples were cut into approximately 3-mm chunks and placed atop tantalum-coated carbon matrix grids for 3 weeks in tissue culture medium to allow egress of tissue-associated lymphocytes. These ‘grid-isolated’ cells were then collected and expanded in tissue culture medium supplemented with recombinant human interleukin-2 (rhIL-2) and recombinant human interleukin-15 (rhIL-15) (Methods) for a further 3 weeks. Thereafter, the ‘skin-expanded’ lymphocytes were cryopreserved before thawing for use in downstream assays. This process greatly enriched for Vδ1^+^ cells, both in proportion and yield, compared with either direct enzymatic disaggregation or a 3-week grid isolation period (Fig. 2b and Extended Data Fig. 2a–c). Note that nowhere in the process were cells exposed to TCR agonists, so their antigen experience reflected that of human T cells in vivo.

We found that a small but substantial proportion of skin-expanded Vδ1^+^ cells expressed PD-1 (Fig. 2c,d), which is consistent with previous descriptions of these cells directly isolated from other epithelial tissues^10,12^. We considered that this may have in part reflected exposure in vivo to TCR ligands, consistent with which 48-h culture of skin-expanded Vδ1^+^ cells in the presence of plate-bound anti-TCR-Vδ1 antibody increased the percentage of cells expressing PD-1, and significantly increased PD-1 expression levels (Fig. 2e).

**Extended Data Fig. 3:**
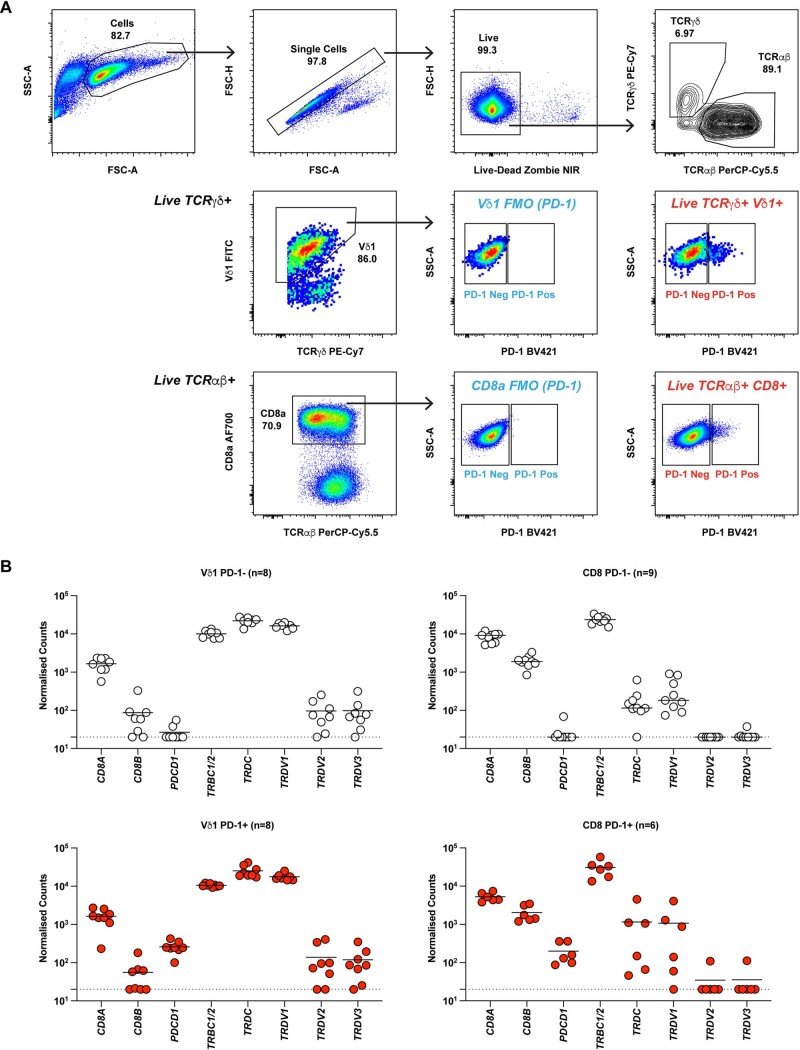
NanoString transcriptomic analysis of skin-derived grid-expanded Vδ1+ and CD8+ T cells. **A**, Representative gating strategy for sorting PD-1- and PD-1 + Vδ1+ and CD8+ T cells from bulk, skin-expanded lymphocytes. PD-1 gate set on fluorescence minus one (FMO) control (blue). PD-1- and PD-1 + Vδ1+ and CD8+ T cells (red) were sorted and lysed in RLT buffer for downstream analysis using the NanoString nCounter Immune Exhaustion Panel. Representative of n = 9 independent donors. **B**, Expression of anticipated canonical lineage genes in each sorted population assessed by NanoString. Dotted line = detection threshold (normalised count = 20). Datapoints represent gene expression in each cell type sorted from individual donors. Source data

We next sorted PD-1^+^ and PD-1^−^Vδ1^+^ cells, and PD-1^+^ and PD-1^−^CD8^+^ T cells derived from identical skin-expanded lymphocyte preparations directly into lysis buffer for transcriptomic analyses (Extended Data Fig. 3a). Thus, where cell numbers were permissive, lysates from approximately 13,000 cells (13,650 ± 2,355 cells, mean ± s.d.) of each sorted population were analyzed by NanoString using the nCounter Immune Exhaustion Panel of 785 genes that was developed based largely on the phenotypes of PD-1^+^CD8^+^ αβ T cells. Gene expression counts were scaled and normalized using nSolver and all samples passed internal quality checks (Methods). Importantly, sorted cell populations expressed the anticipated canonical lineage genes +/− *PDCD1* (Extended Data Fig. 3b). Regarding the use of *TRDV1* as a surrogate marker of Vδ1^+^ cells (considered above), there was some expression of *TRDV1* by sorted CD8^+^ T cells, but it was demonstrably orders of magnitude less than in sorted Vδ1^+^ cells (Extended Data Fig. 3b).

**Extended Data Fig. 4:**
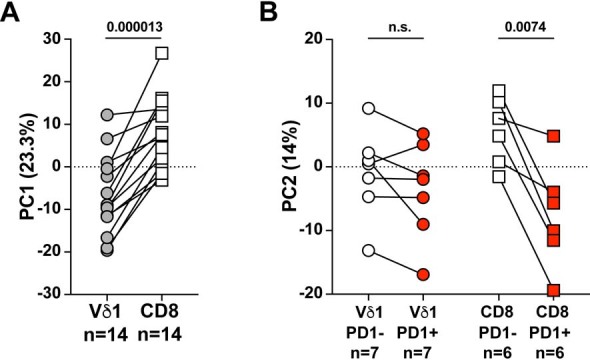
PD-1 expression on Vδ1+ cells is associated with distinct biology compared to its expression on CD8+ T cells. **A**, PC1 from Fig. 2F plotted by cell type. Datapoints paired by PD-1 status and donor (n = 14 pairs). Paired t test. **B**, PC2 from Fig. 2G plotted by cell type and PD-1 status and paired by donor (Vδ1 n = 7 pairs, CD8 n = 6 pairs). Paired t test. All P values presented are two-sided. n.s. = not significant. Source data

We performed a principal component analysis (PCA) based on the normalized counts of all 757 genes, excluding lineage-defining and housekeeping genes (Methods), and observed a clear discrimination on principal component 1 between Vδ1^+^ and CD8^+^ T cells (Fig. 2f,g and Extended Data Fig. 4a). Given that these cells have previously been shown to display comparable overall gene expression profiles by RNA-seq^23,39^, their clear segregation when assessed by a targeted immune exhaustion panel was particularly striking. Likewise, whereas principal component 2 could segregate CD8^+^ T cells according to PD-1 status (as would be wholly expected for a gene panel based on αβ T cell exhaustion), this was conspicuously not the case for Vδ1^+^ cells, which showed intermixing of PD-1^+^ and PD-1^−^ populations (Fig. 2f,g and Extended Data Fig. 4b). Taken together, these data suggest that the status of PD-1^+^Vδ1^+^ cells cannot be described by the molecular phenotype of colocated PD-1^+^CD8^+^ T cells.

In addition to denoting CD8^+^ T cells transiting toward exhaustion, PD-1 is also implicated in the residency and long-term survival of CD8^+^ T cells within tissues, with a recent study suggesting that this might also be true in γδ T cells^12^. This is notable because the tissue-resident phenotype has been increasingly implicated in effective cancer immunosurveillance and response to anti-PD-1 and anti-PD-L1 therapy^23,40–42^. Thus, we directed our analyses toward an established 31-gene signature of human tissue-resident T cells^43^, of which 13 genes were detected by the chosen NanoString panel.

**Fig. 3:**
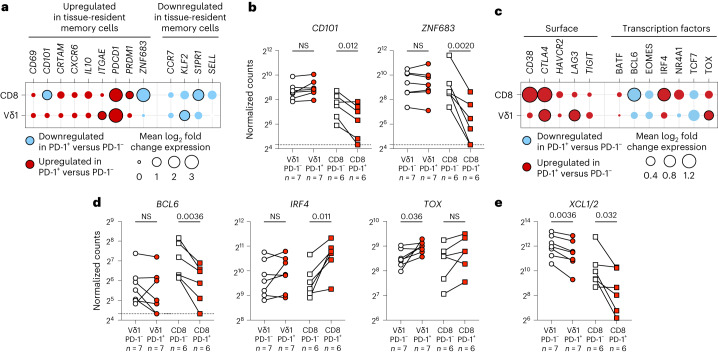
Expression of PD-1 marks a transcriptional program of tissue residency and survival in Vδ1^+^ cells, which is distinct from its associations in CD8^+^ T cells. **a**, Differential expression of genes associated with a tissue-resident memory phenotype in human T cells in sorted skin-expanded lymphocytes, plotted as the mean log_2_ fold change between PD-1^+^ and PD-1^−^Vδ1^+^ and CD8^+^ T cells (Vδ1^+^*n* = 7 paired independent donors; CD8^+^*n* = 6 paired independent donors). The color denotes directionality and the size of the circle denotes the fold change. Empty circles plotted where no samples within a cell type had detectable counts above the threshold (for example, *SELL* in Vδ1^+^ cells) are shown. Paired ratio *t*-test. The black border denotes *P* < 0.05. **b**, Gene expression of *CD101* and *ZNF683* in Vδ1^+^ (left) and CD8^+^ T (right) cells plotted according to PD-1 status. Data points are paired according to the independent donors. Paired ratio *t*-test. The dotted line signifies the detection threshold (normalized count = 20). **c**, Differential expression of genes encoding the surface markers of T cell exhaustion and transcription factors implicated in T cell survival, stemness and exhaustion in sorted skin-expanded lymphocytes. Plotted as the mean log_2_ fold change between PD-1^+^ and PD-1^−^Vδ1^+^ and CD8^+^ T cells (Vδ1^+^, *n* = 7 paired independent donors; CD8^+^, *n* = 6 paired independent donors). The color denotes directionality and the size of the circle denotes the fold change. Paired ratio *t*-test. The black border denotes *P* < 0.05. **d**, Gene expression of *BCL6*, *IRF4* and *TOX* in Vδ1^+^ (left) and CD8^+^ T (right) cells plotted according to PD-1 status. **e**, Gene expression of *XCL1* and *XCL2* in Vδ1^+^ (left) and CD8^+^ T (right) cells plotted according to PD-1 status. The data points are paired according to the independent donors. Paired ratio *t*-test. The dotted line signifies the detection threshold (normalized count = 20). All *P* values presented are two-sided. Source data

Assessing these 13 genes, we found that PD-1^+^Vδ1^+^ and PD-1^+^CD8^+^ T cells were both enriched for the tissue-resident phenotype when compared to their PD-1^−^ counterparts (Fig. 3a). Specifically, both PD-1^+^Vδ1^+^ and PD-1^+^CD8^+^ T cells showed consistent trends defined by the upregulation or maintenance of some canonical tissue residence and homing genes, and downregulation of tissue egress genes (Fig. 3a). Nevertheless, consistent with the evidence (above) that the molecular phenotype of PD-1^+^Vδ1^+^ cells is distinct from that of colocated PD-1^+^CD8^+^ T cells, some genes demonstrated noncongruent regulation across the two cell types (Fig. 3a,b). For example, *CD101*, a marker of tissue residence, was significantly downregulated in PD-1^+^CD8^+^ T cells compared to their PD-1^−^ counterparts but was maintained in PD-1^+^Vδ1^+^ cells (Fig. 3b). A further illustration was provided by *PRDM1* (encoding BLIMP-1) and *ZNF683* (encoding HOBIT). While both genes are linked to tissue residence^44^, *PRDM1* is more strongly implicated in T cell exhaustion^45–47^ while *ZNF683* is associated with T cell competence and survival^48–50^. In this context, *PRDM1* was significantly upregulated and *ZNF683* was significantly downregulated in PD-1^+^CD8^+^ T cells compared to PD-1^−^CD8^+^ T cells, whereas their expression levels across PD-1^+^ and PD-1^−^Vδ1^+^ cells were conspicuously comparable (Fig. 3a,b).

Further evidence that PD-1^+^Vδ1^+^ cells are only partial phenocopies of colocated PD-1^+^CD8^+^ T cells came from an assessment of genes encoding surface receptors known to be enriched in terminally exhausted CD8^+^ T cells, and of transcription factors strongly implicated in the self-renewal, survival or terminal exhaustion of the PD-1^+^CD8^+^ T cell subsets^51^. PD-1^+^Vδ1^+^ and PD-1^+^CD8^+^ T cells were both enriched for the transcripts of the surface receptors associated with exhaustion when compared to their PD-1^−^ counterparts (Fig. 3c). Likewise, both PD-1^+^Vδ1^+^ and PD-1^+^CD8^+^ T cells upregulated *TOX*, albeit only as a trend in the latter (Fig. 3c,d). By contrast, PD-1^+^CD8^+^ T cells displayed clear downregulation of *BCL6*, a transcription factor shown to repress T cell exhaustion and promote T cell stemness and self-renewal^52^, as well as upregulation of the transcription factor *IRF4*, which promotes T cell exhaustion^53^ (Fig. 3c,d). Conversely, PD-1^+^ and PD-1^−^Vδ1^+^ cells showed comparable expression of *BCL6* and *IRF4* (Fig. 3c,d). Moreover, expression of lymphotactin (*XCL1*), a chemokine consistently implicated in the self-renewal and survival of stem-like, pre-exhausted T cells^7,9,54^, was greatly reduced in PD-1^+^CD8^+^ T cells relative to PD-1^−^CD8^+^ T cells but was only modestly decreased in PD-1^+^Vδ1^+^ cells. Indeed, the expression of *XCL1* in PD-1^+^Vδ1^+^ cells (median normalized count = 2,860) was nearly a magnitude greater than in PD-1^+^CD8^+^ T cells (median normalized count = 330) (Fig. 3e). Nonetheless, PD-1^+^Vδ1^+^ cells could not be easily classified as stem-like PD-1^+^ T cells because they did not upregulate the transcription factor TCF-1 (*TCF7*), which has been consistently linked to stemness in PD-1^+^CD8^+^ T cells^7–9^ (Fig. 3c).

### PD-1^+^Vδ1^+^ cells remain functional

**Fig. 4:**
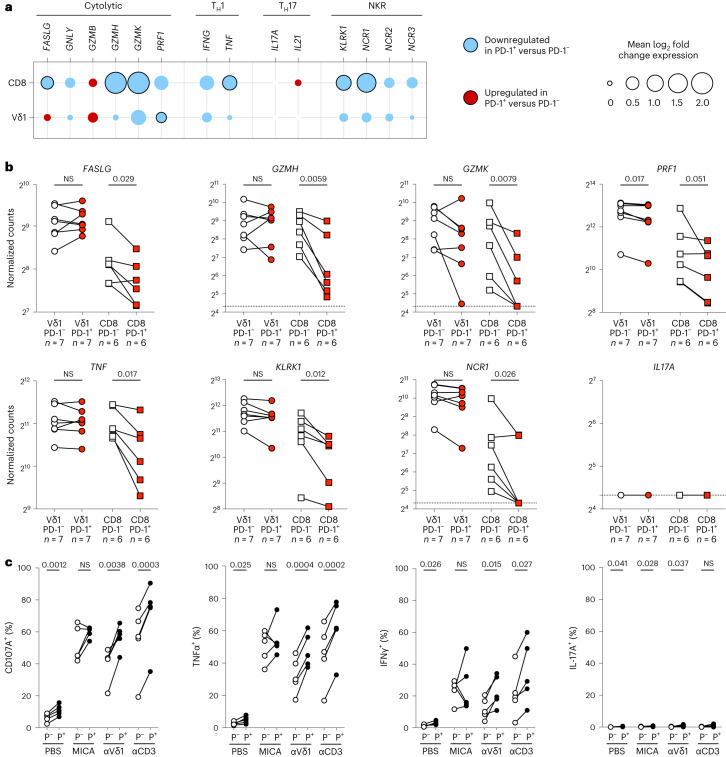
PD-1^+^Vδ1^+^ cells are functionally competent. **a**, Differential expression of genes associated with pro-tumor and anti-tumor T cell functions in sorted skin-expanded lymphocytes, plotted as the mean log_2_ fold change between PD-1^+^ and PD-1^−^ Vδ1^+^ and CD8^+^ T cells (Vδ1^+^, *n* = 7 paired independent donors; CD8^+^, *n* = 6 paired independent donors). The color denotes directionality and the size of the circle denotes the fold change. Empty circles plotted where no samples within a cell type had detectable counts above threshold are shown. Paired ratio *t*-test. The black border denotes *P* < 0.05. **b**, Gene expression of *FASLG*, *GZMH*, *GZMK*, *PRF1*, *TNF*, *KLRK1*, *NCR1* and *IL17A* in Vδ1^+^ (left) and CD8^+^ T (right) cells plotted according to PD-1 status. The data points are paired according to the independent donors. Paired ratio *t*-test. The dotted lines denote the limit of detection (normalized count = 20). **c**, Summary flow cytometry data of surface CD107A and intracellular TNF, IFNγ and IL-17A staining of in vitro-activated, skin-expanded PD-1^−^ (P^−^, white circle) and PD-1^+^ (P^+^, black circle) Vδ1^+^ cells. Cells were activated in vitro with PBS (negative control), plate-bound MICA, plate-bound anti-TCR-Vδ1 or plate-bound anti-CD3 as indicated. The data points are paired according to *n* = 5 independent donors. Paired *t*-test. Source data

We next focused on genes linked to effective γδ T cell cancer immunosurveillance in both murine models and clinical disease, namely those encoding cytotoxic molecules, type 1 helper T (T_H_1) cytokines and activating natural killer receptors (NKRs)^15,24^. We also looked at genes encoding type 17 helper T (T_H_17) cytokines, which have been linked to cancer promotion by γδ T cells, albeit predominantly in murine models^15,24^. Compared to PD-1^−^ memory CD8^+^ T cells, PD-1^+^CD8^+^ T cells have reduced cytotoxic and T_H_1 effector functions^55,56^; indeed, this was illustrated in the skin-expanded T cell cultures. Thus, PD-1^+^CD8^+^ T cells showed markedly reduced expression of *FASLG* (encoding the Fas ligand), *GZMH* and *GZMK* (encoding two types of granzyme), *PRF1* (encoding perforin), *TNF* (encoding tumor necrosis factor (TNF)), as well as *KLRK1* and *NCR1* (encoding the activating NKRs, NKG2D and NKp46, respectively) (Fig. 4a,b). By contrast, PD-1^+^Vδ1^+^ cells displayed largely comparable expression of most of these genes relative to their PD-1^−^ counterparts. Although *PRF1* was significantly downregulated in PD-1^+^Vδ1^+^ cells, its expression in these cells (median normalized count = 5,102) was over four times greater than in PD-1^+^CD8^+^ T cells (median normalized count = 1,198) (Fig. 4a,b). Notably, cytotoxic and T_H_1 effector functions, as well as activating NKRs, have consistently been linked to patient-beneficial cancer immunosurveillance^57–59^; Vδ1^+^ cells generally expressed higher amounts of these genes compared to CD8^+^ T cells (Fig. 4b). Additionally, we found no evidence of *IL17A* expression, which is consistent with multiple studies demonstrating that human Vδ1^+^ cells producing IL-17 are rare^10,11,23,36,60,61^. Thus, the expression of PD-1 by tissue-associated Vδ1^+^ cells occurred against a background of the cells’ strong and selective functional potentials.

**Extended Data Fig. 5:**
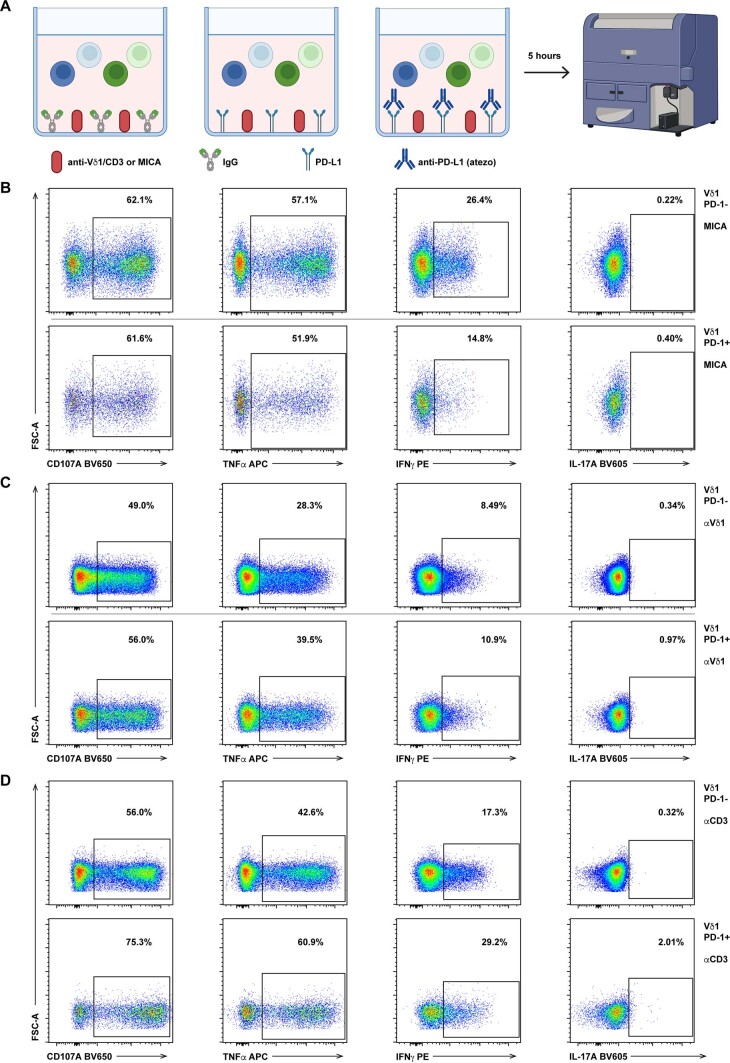
In vitro activation of skin-expanded Vδ1+ cells. **A**, Schema of in vitro activation assay. Plates were coated with anti-TCR-Vδ1, anti-CD3 or MICA along with either mouse IgG (control) or rhPD-L1 overnight. Atezolizumab was added 30 minutes prior to addition of skin-expanded lymphocytes to relevant wells. Skin-expanded lymphocytes were seeded in the presence of brefeldin A and incubated for 5 hours at 37^o^C before staining for flow cytometry and acquisition. Representative flow cytometry plots of surface CD107A, and intracellular TNFα, IFNγ and IL-17A staining after activation with **B**, MICA, **C**, anti-TCR-Vδ1 or **D**, anti-CD3. Gated either PD-1- or PD-1 + Vδ1+ cells. Representative of n = 5 independent donors.

To assess the realization of these functional potentials, we measured the cytokine production and cytotoxic degranulation of skin-expanded PD-1^+^Vδ1^+^ cells in response to activation in vitro. While previous studies demonstrated that these cells retain function comparable to their PD-1^−^ counterparts, they have largely done so in the context of phorbol myristate acetate and ionomycin activation^12,14^, which bypasses the proximal physiological signaling modalities that regulate Vδ1^+^ cells in vivo. Thus, we exposed skin-expanded Vδ1^+^ cells either to agonists for the TCR, namely plate-bound anti-TCR-Vδ1 or anti-CD3, or to agonists for the activating cytotoxic NKR, NKG2D (encoded by *KLRK1*), namely recombinant MHC class I polypeptide-related sequence A (MICA), a stress-associated ligand upregulated in many cancers (Extended Data Fig. 5a). In response to NKG2D stimulation (recombinant MICA), skin-expanded PD-1^+^Vδ1^+^ cells produced interferon-γ (IFNγ) and TNF and degranulated (measured using surface CD107A staining) to comparable levels as PD-1^−^Vδ1^+^ cells (Fig. 4c and Extended Data Fig. 5b). However, PD-1^+^Vδ1^+^ cells were more responsive to TCR stimulation (anti-TCR-Vδ1 or anti-CD3) compared to their PD-1^−^ counterparts, which is consistent with some data in a recent study of human colorectal cancer (CRC)^13^ (Fig. 4c and Extended Data Fig. 5c,d). Furthermore, we again found little evidence of IL-17 production by Vδ1^+^ cells, which is in agreement with our transcriptomic analysis and with previous studies (Fig. 4c and Extended Data Fig. 5b–d)^10,11,23,36,61^.

### Vδ1^+^ cells can be regulated by PD-1 and derepressed by CPI

**Fig. 5:**
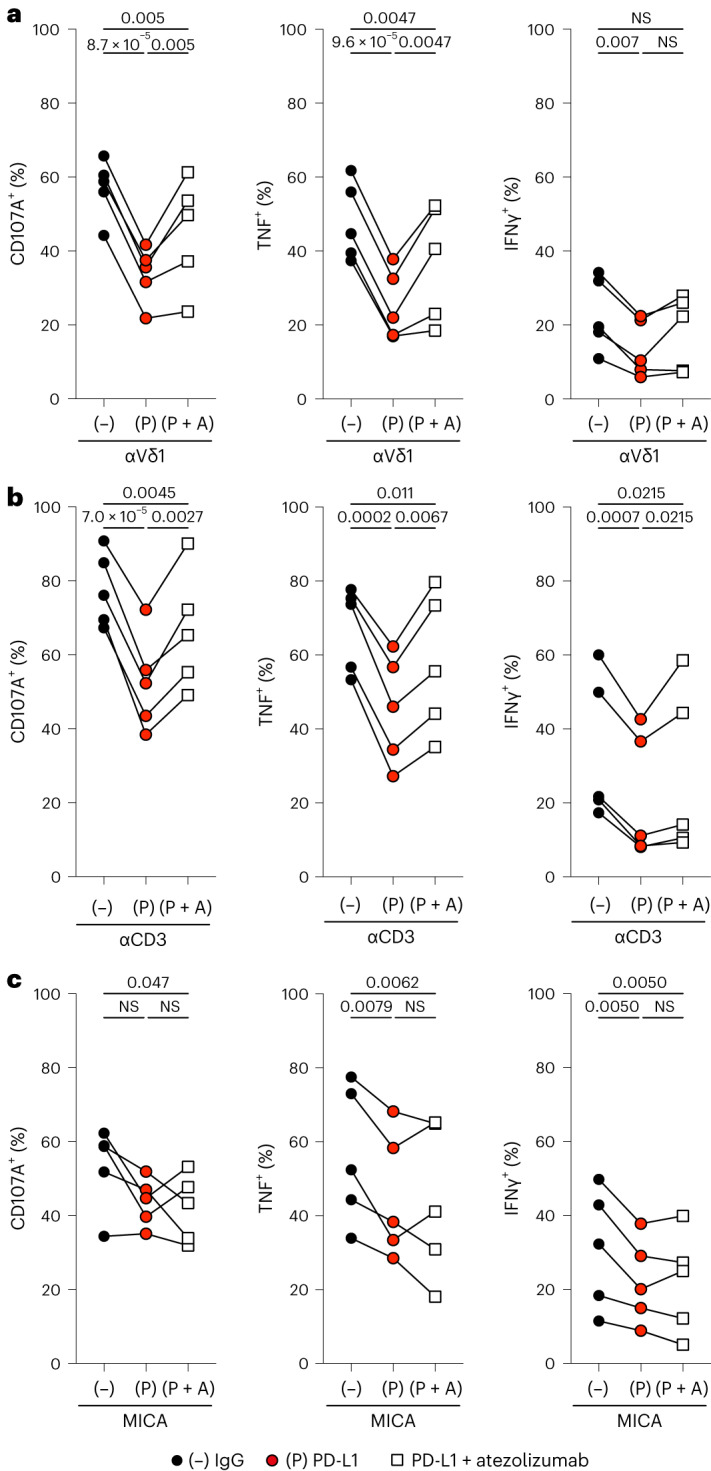
PD-1^+^Vδ1^+^ cells can be regulated by PD-1 engagement and derepressed by therapeutic CPI. **a**, Summary flow cytometry data of surface CD107A, and intracellular TNF and IFNγ staining, in PD-1^+^Vδ1^+^ cells activated in vitro with plate-bound anti-TCR-Vδ1 antibody in the presence of control plate-bound mouse IgG (−), rhPD-L1 (P) or rhPD-L1 and atezolizumab (P + A). **b**, Summary flow cytometry data of surface CD107A, and intracellular TNF and IFNγ staining in PD-1^+^Vδ1^+^ cells activated in vitro with plate-bound anti-CD3 antibody in the presence of control plate-bound mouse IgG (−), rhPD-L1 (P) or rhPD-L1 and atezolizumab (P + A). **c**, Summary flow cytometry data of surface CD107A, and intracellular TNF and IFNγ staining, in PD-1^+^Vδ1^+^ cells activated in vitro with plate-bound MICA in the presence of control plate-bound mouse IgG (−), rhPD-L1 (P) or rhPD-L1 and atezolizumab (P + A). A repeated measures one-way ANOVA followed by a Holm–Šídák’s multiple comparisons test was used. The data points are paired according to *n* = 5 independent donors. Adjusted *P* values are shown. All *P* values presented are two-sided. Source data

Despite multiple studies proposing that PD-1 may regulate human Vδ1^+^ cell responses, no direct evidence for this has been provided^13,14,23^. To redress this, we activated skin-expanded PD-1^+^Vδ1^+^ cells in the presence of recombinant human PD-L1 (rhPD-L1) (Extended Data Fig. 5a) and found that this markedly attenuated the production of effector cytokines and degranulation, particularly in the context of TCR stimulation (Fig. 5a,b). The attenuation of TCR-dependent activation is consistent with the well-described mechanism of action for PD-1 in the context of αβ T cell repression^25^. Nonetheless, we also observed a more modest attenuation of PD-1^+^Vδ1^+^ cell responses to innate NKG2D stimulation (Fig. 5c).

**Extended Data Fig. 6:**
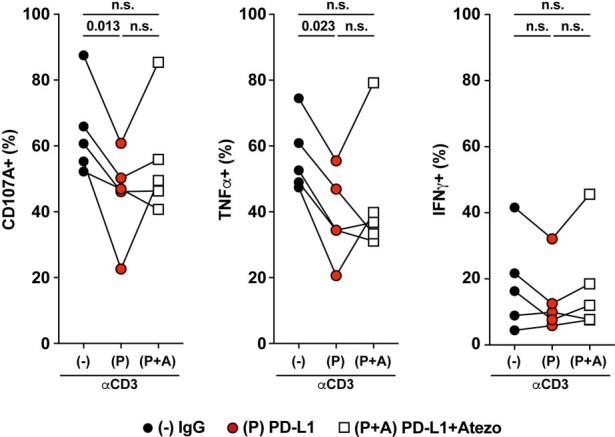
PD-1+ CD8+ T cells are suppressed after PD-1 engagement with variable de-repression by therapeutic CPI. Summary flow cytometry data of surface CD107A, and intracellular TNFα and IFNγ staining in skin-expanded PD-1+ CD8+ T cells activated in vitro with plate-bound anti-CD3 in the presence of control plate-bound mouse IgG (-), rhPD-L1 (P), or rhPD-L1 and atezolizumab (P + A). Repeated measures one-way ANOVA followed by Holm-Sidak’s multiple comparisons test. Datapoints paired by n = 5 independent donors. Adjusted P values indicated. All P values presented are two-sided. n.s. = not significant. Source data

To relate these observations to the setting of CPI therapies, we tested if PD-1/PD-L1 blockade could rescue the effector functions of activated PD-1^+^Vδ1^+^ cells exposed to rhPD-L1 (Extended Data Fig. 5a). We indeed observed partial rescue of function in the presence of the clinically approved anti-PD-L1 antibody atezolizumab, predominantly in the context of TCR activation (Fig. 5a–c). By contrast, while skin-expanded PD-1^+^CD8^+^ T cells activated with anti-CD3 could be inhibited by rhPD-L1 as expected, the rescue of function by atezolizumab was more variable and less apparent compared with PD-1^+^Vδ1^+^ cells (Extended Data Fig. 6).

Finally, to establish if these traits are retained by PD-1^+^Vδ1^+^ cells in the tumor microenvironment (TME), we repeated our in vitro functional assay using tumor-derived T cells. Primary melanomas rarely exceed a few millimeters in depth and routinely yield negligible material surplus to clinical diagnostic needs. Akin to skin and melanomas, steady-state lung tissue and non-small-cell lung cancer (NSCLC) also harbor a population of tissue-resident PD-1^+^Vδ1^+^ cells^23^. Likewise, the presence of Vδ1^+^ cells in NSCLC is also associated with favorable clinical outcomes^23,36^. Moreover, anti-PD-1 and anti-PD-L1 CPI therapies are also effective in the treatment of NSCLC^62^. Thus, we used the grid approach to expand tumor-infiltrating lymphocytes (TILs) (Fig. 2a) from treatment-naive, surgically resected primary NSCLC for functional testing (Supplementary Table 1). Given the limited primary material, NSCLC-expanded TILs were activated using only plate-bound anti-CD3 and MICA.

**Fig. 6:**
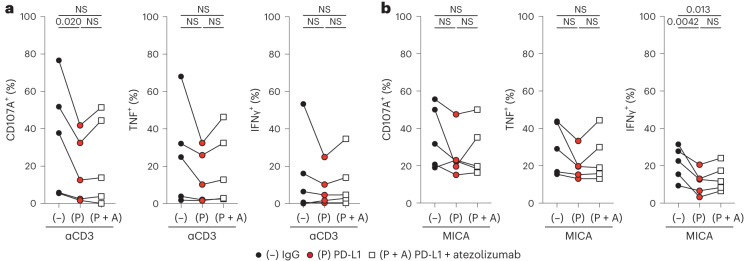
Primary tumor-derived PD-1^+^Vδ1^+^ cells are variably regulated by PD-1 engagement and derepressed by therapeutic CPI. **a**, Summary flow cytometry data of surface CD107A, and intracellular TNF and IFNγ staining, in NSCLC-expanded PD-1^+^Vδ1^+^ cells activated in vitro with plate-bound anti-CD3 antibody in the presence of control plate-bound mouse IgG (−), rhPD-L1 (P) or rhPD-L1 and atezolizumab (P + A). **b**, Summary flow cytometry data of surface CD107A, and intracellular TNF and IFNγ staining, in NSCLC-expanded PD-1^+^Vδ1^+^ cells activated in vitro with plate-bound MICA in the presence of control plate-bound mouse IgG (−), rhPD-L1 (P) or rhPD-L1 and atezolizumab (P + A). A repeated measures one-way ANOVA followed by a Holm–Šídák’s multiple comparisons test was used. The data points are paired according to *n* = 5 independent donors. Adjusted *P* values are shown. All *P* values presented are two-sided. Source data

**Extended Data Fig. 7:**
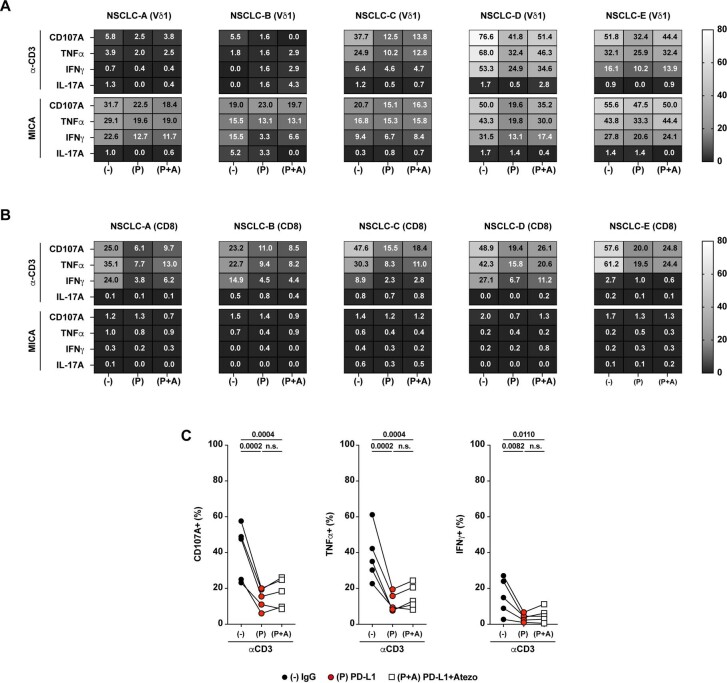
Functional regulation of primary NSCLC-expanded Vδ1+ and CD8 T+ cells by PD-1. **A**, Donor level flow cytometry staining of surface CD107A, and intracellular TNFα, IFNγ and IL-17A in NSCLC-expanded PD-1+ Vδ1+ cells activated in vitro with plate-bound anti-CD3 (top) and MICA (bottom) in the presence of control plate-bound mouse IgG (-), rhPD-L1 (P), or rhPD-L1 and atezolizumab (P + A). **B**, Donor level flow cytometry staining of surface CD107A, and intracellular TNFα, IFNγ and IL-17A in PD-1+ NSCLC-expanded CD8+ T cells activated in vitro with plate-bound anti-CD3 (top) and MICA (bottom) in the presence of control plate-bound mouse IgG (-), rhPD-L1 (P), or rhPD-L1 and atezolizumab (P + A). Cell values and shading denote percentage of parent gate (PD-1+ Vδ1 cells or PD-1+ CD8+ T cells) positive for stain. **C**, Summary flow cytometry data of surface CD107A, and intracellular TNFα and IFNγ staining in NSCLC-expanded PD-1+ CD8+ T cells activated in vitro with plate-bound anti-CD3 antibody in the presence of control plate-bound mouse IgG (-), rhPD-L1 (P), or rhPD-L1 and atezolizumab (P + A). Repeated measures one-way ANOVA followed by Holm-Sidak’s multiple comparisons test. Datapoints paired by n = 5 independent donors. Adjusted P values indicated. All P values presented are two-sided. n.s. = not significant. Source data

We observed a broad spectrum of degranulation and T_H_1 cytokine production after anti-CD3 activation by NSCLC-expanded PD-1^+^Vδ1^+^ cells, probably reflecting a more heterogeneous state of these cells in the TME. Nonetheless, in cases where cells were responsive to TCR activation, we observed clear inhibition by PD-L1 and partial derepression of this with atezolizumab (Fig. 6a and Extended Data Fig. 7a). Intriguingly, NSCLC-expanded PD-1^+^Vδ1^+^ cells from all patients could be activated using plate-bound MICA, regardless of TCR responsiveness (Fig. 6b and Extended Data Fig. 7a). Consistent with our observations in skin-expanded PD-1^+^Vδ1^+^ cells, the NSCLC-expanded counterparts activated by plate-bound MICA could also be inhibited by PD-L1, albeit with more interdonor variability (Fig. 6b and Extended Data Fig. 7a). Likewise, addition of atezolizumab demonstrated variable derepression in the context of MICA activation, potentially reflecting some degree of TCR activation, for example, as a legacy of the TME (Fig. 6b and Extended Data Fig. 7a). By comparison, NSCLC-expanded PD-1^+^CD8^+^ T cells activated by anti-CD3 were highly sensitive to PD-L1 inhibition with only minimal rescue of function in the presence of atezolizumab (Extended Data Fig. 7b,c). Thus, human tissue-resident Vδ1^+^ cells, including those derived from the TME, are functionally competent cells that can be regulated by PD-1 engagement and derepressed by CPI.

## Discussion

Many cancers with reduced visibility to αβ T cell immunosurveillance, for example, via loss of MHC or low mutational burdens, nonetheless remain exquisitely sensitive to CPI therapy^63,64^. Given their intratumoral presence, expression of PD-1 (refs. ^10,11,36^) and capacity to kill transformed cells independently of TCR (neo)antigen activation, multiple studies suggested Vδ1^+^ cells as effector targets of CPI^13,14,23^. However, such considerations and their promising potential to rationally guide clinical translation remain speculative until the impact of PD-1 expression on these cells has been established. This has been addressed in this study.

By using an in vitro explant system to isolate sufficient numbers of PD-1^+^Vδ1^+^ cells from human tissues and tumors, we now provide evidence that human PD-1^+^Vδ1^+^ cells are unlike their CD8^+^ αβ T cell counterparts in maintaining the expression of key genes related to tissue residence and effector potentials. Furthermore, we show that PD-1^+^Vδ1^+^ cells remain functionally responsive to physiologically relevant innate (NKR) and adaptive (TCR) immune agonists, and that these cells can be regulated by PD-1 engagement and derepressed by PD-1/PD-L1 blockade. These observations probably reflect the physiological niche γδ T cells occupy as local immune sentinels of tissue health in real time^65^. Importantly, we present evidence that the intratumoral presence of Vδ1^+^ cells is a predictor of therapeutic response in patients with melanoma receiving anti-PD-1 and anti-PD-L1 therapy, particularly in the setting of low neoantigen loads where αβ T cell surveillance is most likely compromised.

Human Vδ1^+^ cells are prominent within barrier tissues where they maintain tissue integrity against myriad challenges, including cancer^10,12,23,29^. Their innate-like biology means that cells must combine competence to make rapid effector functions in response to inevitable perturbations imposed by the external environment, with tissue-based immunoregulation to mitigate autoinflammation. Moreover, while αβ T cells can be continually recruited into tissues throughout life^66^, it is unclear if this applies to Vδ1^+^ cells, which probably populate tissues during limited developmental windows^37,67,68^. Thus, while differentiation toward terminal exhaustion may be an effective fail-safe mechanism to limit αβ T cell autoinflammation, it may not be appropriate in the context of tissue-associated Vδ1^+^ cells. Note that this is not to suggest that there are not PD-1^+^Vδ1^+^ cells that perfectly phenocopy PD-1^+^CD8^+^ αβ T cells and vice versa, but the balance of these subtypes may be very different within the aggregate PD-1^+^ γδ and αβ T cell populations, respectively.

In this study, the priority was to assess population-level phenotypes because those will provide the aggregate responses to PD-L1 regulation and CPI in persons in vivo, albeit that follow-up single-cell resolution approaches may identify more specific targets of CPI within the Vδ1^+^ cell population. Indeed, the variable responses observed in vitro, both in skin-expanded and more notably in NSCLC-expanded PD-1^+^Vδ1^+^ cells, allude to a degree of heterogeneity of Vδ1^+^ cells in vivo as recently demonstrated in the context of CRC^36^. We acknowledge several limitations of our study. While the association of intratumoral *TRDV1* with response to CPI therapy in melanoma is consistent with reports in other tumor types^14,23^, we are cognizant of the relatively small sample size of our study, particularly in the context of neoantigen burden. Likewise, it was not possible to directly visualize the presence of Vδ1^+^ cells in tumor specimens because no antibodies suitable for use on formalin-fixed paraffin-embedded (FFPE) sections exist. Nonetheless, *TRDV1* transcripts and composite gene signatures are widely accepted surrogates for these cells^11,14,22,23,36^. That neither *TRBC2*, *CD4* nor *CD8B* expression within melanomas was predictive of a response to CPI therapies also supports a bona fide association of response with Vδ1^+^ cells.

The biology and functional relevance of PD-1 expression in Vδ1^+^ cells in tissues and tumors merit further investigation. For example, given that PD-1-dependent suppression of effector function was most profound in the context of TCR stimulation, an obvious question arises as to which ligand(s) TCRVδ1 recognizes within tumors and tissues. Our formal demonstration that Vδ1^+^ cells can be suppressed by PD-1 and partially derepressed by PD-1/PD-L1 blockade supports the utility of these cells as a predictive biomarker and may open potentially curative immunotherapies to patients who would otherwise not be eligible for treatment based on mutational burden alone^69^. However, this does not diminish the importance of αβ T cells, nor that of other immune cells expressing PD-1 (for example, NK cells, macrophages)^70,71^, which most likely act in concert to seal off multiple paths of the immune evasion arising during tumor evolution.

While our expansion protocol has enabled the preclinical study of these rare cells, it clearly falls short of the requisite yields for ACT. Nonetheless, our findings may guide the development of clinical trials of Vδ1^+^ ACTs. Two major limitations of contemporary ACTs for the treatment of solid cancers have been poor tumor homing and persistence^72,73^. Germane to this, the enrichment of tissue homing and residence genes and maintenance of persistence and survival genes in PD-1^+^Vδ1^+^ cells relative to their PD-1^−^ counterparts may prove advantageous in the setting of solid cancer ACT^74^.

## Methods

Research involving human samples complied with all relevant ethical regulations as detailed below. Participants were not financially compensated.

### Human skin samples

Ethical approval for the study was obtained from the Camberwell and St Giles Research Ethics Committee (15/LO/2130, Integrated Research Approval System (IRAS): 169471). After written informed consent was obtained, skin samples that would otherwise have been discarded were obtained from patients undergoing elective plastic and reconstructive surgical procedures. Anatomically, samples were either derived from the breast or abdomen.

### Human NSCLC samples

Human NSCLC samples and clinical data were supplied by the King’s Health Partners Cancer Biobank (REC: 18/EE/0025, IRAS: 240747). All patients provided written informed consent for the collection of tissue excess to diagnostic requirements by the King’s Health Partners Cancer Biobank. All donors of NSCLC tissue were male.

### Melanoma CPI response analysis

Source data were downloaded as supplementary tables from Liu et al.^33^. The Gene Expression Omnibus (GEO) was accessed for the four patient cohorts published by Auslander et al.^32^ (GSE115821), Du et al.^34^ (GSE168204), Hugo et al.^30^ (GSE78220) and Riaz et al.^31^ (GSE91061). Data were downloaded in the NCBI-generated RNA-seq format as TPM. This yielded an initial combined cohort of 322 cases (used to plot Extended Data Fig. 1a). To analyze the immunotherapy responses, cases were dichotomized as either responders (including partial and complete responses) or nonresponders (including stable or progressive disease); cases with missing response data or mixed responses were excluded, thus leaving 316 cases. Cases were further selected for baseline samples (that is, taken before commencing CPI as opposed to during CPI therapy) and for patients receiving CPI regimens, which included anti-PD-1 or anti-PD-L1 (both single-agent or combination therapy), thus leaving 216 cases (Extended Data Fig. 1b). Finally, analysis was restricted to cases in which *TRDV1* transcripts could be detected, thus yielding a cohort of *n* = 127 cases (Fig. 1a). Given the impact of methodology on TMB and neoantigen load estimates (for example, sample preparation (FFPE versus frozen versus fresh), sequencing depth and neoantigen prediction algorithm), we restricted our analysis of responses in the context of neoantigen burden to the largest dataset by Liu et al.^33^ (*n* = 61 based on the above filters) (Fig. 1b,c). Neoantigen burden was based on the total neoantigens from Supplementary Table 1. For the survival analysis, survival was based on progression-free survival (PFS) and a PFS event was based on the ‘progressed’ status in Supplementary Table 1 (ref. ^33^).

### Enzymatic digestion and isolation of skin lymphocytes

To directly isolate skin-resident T lymphocytes immediately ex vivo, we developed a method based on a previously described protocol^75^ and a commercially available human tissue dissociation kit (Whole Skin Dissociation Kit, Human, Miltenyi Biotec). Skin samples were rinsed in sterile PBS and subcutaneous fat was removed using sterile forceps and a scalpel. Tissue fragments were generated using a 3-mm punch biopsy (Kai Medical) and then further divided using sterile forceps and a scalpel into three approximately equally sized pieces. Twelve such pieces were transferred to a gentleMACS C tube (Miltenyi Biotec) and digested according to the Whole Skin Dissociation Kit protocol (Miltenyi Biotec). Enzyme P was omitted to minimize degradation of relevant epitopes for downstream flow cytometry. Samples were then digested at 37 °C and 5% CO_2_ for 3 h. After 3 h, samples were diluted with 500 µl of medium (Roswell Park Memorial Institute (RPMI) 1640 + 10% FCS) and transferred to the gentleMACS dissociator for mechanical tissue dissociation (gentleMACS program h_skin_01). After dissociation, 200 Kunitz units ml^−1^ of DNase I (Merck Millipore) were added and samples were incubated for 15 min at room temperature. The resulting cell suspensions were then filtered through a 100-µM strainer and then a 70-µM strainer. Filtered cells were then washed with PBS + 10 mmol l^−1^ EDTA at 4 °C. Washed cell pellets were resuspended in 1 ml of cold medium (RPMI 1640 + 10% FCS) and passed through a 40-µM strainer before subsequent analyses.

### Grid isolation and expansion of skin lymphocytes and NSCLC TILs

Lymphocytes were expanded from primary human tissues (skin or NSCLC) by adapting an in vitro explant culture system first described by Clark et al.^76^. For human skin, subcutaneous fat was first removed using sterile forceps and a scalpel, and 3-mm fragments were generated using a punch biopsy (Kai Medical). For NSCLC, tumor tissue was cut to approximately 3-mm fragments using sterile forceps and a scalpel. Three fragments (either skin or NSCLC) were placed onto sterile tantalum-coated carbon foam grids (Ultramet) and positioned into the wells of a G-Rex6 plate (Wilson Wolf Manufacturing Corporation). The grid and tissue explant combinations were cultured in 30 ml per well of complete medium (AIM V, l-glutamine, 100 U ml^−1^ penicillin, 100 µg ml^−1^ streptomycin sulfate, 10 µg ml^−1^ gentamicin sulfate, 10% heat-inactivated FCS and 2.5 μg ml^−1^ amphotericin B), supplemented with rhIL-2 (100 IU ml^−1^, Novartis) and rhIL-15 (20 ng ml^−1^, BioLegend) for 21 days at 37 °C and 5% CO_2_. After 3 weeks in culture, grids were removed from the wells using sterile forceps; grid-isolated lymphocytes were subsequently collected using aspiration.

Grid-isolated lymphocytes were washed in PBS and resuspended in R10 medium (RPMI 1640, l-glutamine, 10% heat-inactivated FCS, 100 U ml^−1^ penicillin, 100 μg ml^−1^ streptomycin sulfate, 1× minimal essential medium nonessential amino acids, 50 μM 2-mercaptoethanol, 1 mM sodium pyruvate and 10 mM HEPES) at 1 million cells per ml. Two million grid-isolated lymphocytes were seeded per well into a 24-well plate (VWR). Cultures were supplemented with rhIL-2 (100 IU ml^−1^) and rhIL-15 (20 ng ml^−1^). Cultures were fed three times per week by removing half of the old medium in each well and adding back the same volume of fresh R10 medium supplemented with 2× concentrations of IL-2 (200 IU ml^−1^) and IL-15 (40 ng ml^−1^). Cultures were maintained for 21 days at 37 °C and 5% CO_2_. Cells were evaluated using phase contrast microscopy at every medium change. At confluency, cells were split (1:1 ratio) into fresh R10 medium containing IL-2 (100 IU ml^−1^) and IL-15 (20 ng ml^−1^). After 3 weeks, skin-expanded and NSCLC-expanded lymphocytes were collected, frozen in CryoStor CS10 freezing medium and stored in liquid nitrogen before thawing for subsequent analyses.

### PD-1 upregulation assay

Skin-expanded lymphocytes were thawed and rested overnight in R10 medium supplemented with rhIL-2 (100 IU ml^−1^) and rhIL-15 (20 ng ml^−1^); 48-well tissue culture plates were coated overnight at 4 °C with control mouse IgG2a (1 μg ml^−1^) or anti-TCRVδ1 (clone REA173, 1 μg ml^−1^, Miltenyi Biotec). After overnight coating, plates were washed three times with PBS; skin-expanded lymphocytes were added at 500,000 cells per well in 800 μl of R10 medium supplemented with rhIL-2 (100 IU ml^−1^) and rhIL-15 (20 ng ml^−1^). After 48 h, cells were collected and stained for flow cytometry.

### In vitro activation assays

Skin-expanded and NSCLC-expanded lymphocytes were thawed and rested overnight in R10 medium supplemented with rhIL-2 (100 IU ml^−1^) and rhIL-15 (20 ng ml^−1^); 96-well tissue culture plates were coated overnight at 4 °C with mouse IgG2a (30 μg ml^−1^, equimolar control for rhPD-L1 (BioLegend)) or rhPD-L1 (10 μg ml^−1^; BioLegend) along with either recombinant human MICA-Fc protein (10 μg ml^−1^, Bio-Techne), anti-Vδ1 (clone REA173, 10 μg ml^−1^, Miltenyi Biotec) or anti-CD3 (clone OKT3, 1 μg ml^−1^, BioLegend). After overnight coating, plates were washed three times with PBS and incubated for 30 min at 37 °C with 100 μl R10 medium alone or 100 μl R10 medium containing atezolizumab (Tecentriq, 60 μg ml^−1^, Roche) per well. After 30 min, overnight-rested, skin-expanded and NSCLC-expanded lymphocytes were seeded at 200,000 cells in 100 μl of medium added directly to each well. Brefeldin A was added at a final concentration of 5 μg ml^−1^ and anti-CD107A was added at a final concentration of 1:400 (v/v). Plates were incubated at 37 °C and 5% CO_2_ for 5 h before collection and staining for flow cytometry.

### Flow cytometry and fluorescence-activated cell sorting

Cells were washed twice in PBS to remove traces of serum before staining with Zombie NIR viability dye at 1:1,000 dilution in PBS for 15 min at room temperature. Cells for downstream NanoString transcriptomics were stained for 20 min at 4 °C. Cells were then stained with an antibody cocktail against surface markers for 20 min at 4 °C in fluorescence-activated cell sorting (FACS) buffer, washed twice and then resuspended in FACS buffer (PBS + 1 mM EDTA + 2% FCS v/v) before acquisition on a BD LSRFortessa cell analyzer or sorting on a BD FACSAria Fusion cytometer. For intracellular cytokine staining, samples were fixed after surface staining using the BD CellFIX tissue reagent followed by two washes with Perm/Wash buffer (BioLegend) and intracellular staining with an antibody cocktail for 30 min at 4 °C in Perm/Wash buffer. After 30 min, samples were washed twice with Perm/Wash buffer and resuspended in FACS buffer before acquisition on a BD LSRFortessa cell analyzer running BD FACSDiva v.9.0. FCS3.0 files were analyzed using FlowJo v.10.

### Ki-67 assay

Grid-isolated and skin-expanded lymphocytes were collected at the end of grid isolation and on day 14 of the 21-day expansion protocol (see above), respectively. Cells were stained for surface lineage markers as described above before fixation and permeabilization using the Intracellular Fixation and Permeabilization Buffer Set (eBioscience). Cells were then stained with anti-Ki-67 Brilliant Violet 421 (BV421) or an isotype control (BioLegend) for 30 min at room temperature. The Ki-67 MFI index was calculated as the ratio between the BV421 MFI of the entire anti-Ki-67 BV421-stained Vδ1^+^ cell population and the BV421 MFI of the entire isotype control-stained Vδ1^+^ cell population.

### NanoString

PD-1^+^ and PD-1^−^Vδ1^+^ and PD-1^+^ and PD-1^−^CD8^+^ T cells were sorted using FACS from skin-expanded lymphocytes (Extended Data Fig. 3a). Cells were sorted into R10 medium at 4 °C and subsequently pelleted and lysed using the RLT lysis buffer (QIAGEN). Lysates from an average of approximately 13,000 cells (13,650 ± 2,355 cells, mean ± s.d.) per sorted population were hybridized to the nCounter Immune Exhaustion Panel to profile 785 human genes at 65 °C overnight (NanoString Technologies). Hybridized samples were processed on an nCounter Prep Station and data were collected on an nCounter Digital Analyzer (NanoString Technologies), according to the manufacturer’s instructions. Raw data were imported into nSolver v.4.0 (NanoString Technologies) for data quality checks, background thresholding, scaling and normalization. Raw counts were scaled based on the geometric mean of all positive control probes. Gene expression normalization (normalized counts) was performed relative to the geometric mean of all housekeeping genes included in the panel. The lower limit of detection was set at a normalized count of 20, which encompassed all negative control reference probes. All samples passed the default nSolver v.4.0 quality checks after this process. Normalized counts were used for all subsequent analyses. Lineage genes directly linked to the FACS sorting strategy (*PDCD1*, *CD8A*, *CD8B*, *TRAC*, *TRBC1* and *TRBC2*, *TRDC*, *TRDV1*, *TRDV2*, *TRDV3*, *TRGC1*, *TRGC2*, *TRGV2*, *TRGV3* and *TRGV4*, *TRGV5*, *TRGV8* and *TRGV9*) and the 12 internal reference housekeeping genes were excluded from the PCA (Fig. 2f,g). Differential gene expression analyses were restricted to paired samples.

### Statistics and reproducibility

No statistical method was used to predetermine sample size. Samples were chosen based on the availability of material with no other selection criteria. No samples were excluded from the analysis. The experiments were not randomized and investigators were not blinded to the conditions of the experiments. Statistical analyses were performed using JMP Pro v.17 and Prism v.9.5.0 (GraphPad Software). The statistical tests used are shown in the figure legends. All statistical tests conducted, and *P* values presented, were two-sided where relevant. Where parametric tests were used, data distribution was assumed to be normal, but this was not formally tested. Significant values (*P* < 0.05) are indicated for all figures where relevant.

### Reporting summary

Further information on research design is available in the Nature Portfolio Reporting Summary linked to this article.

### Supplementary information

Reporting Summary

Supplementary Table 1 Supplementary Table 1.

### Source data

Source Data Fig. 1 Statistical source data.

Source Data Fig. 2 Statistical source data.

Source Data Fig. 3 Statistical source data.

Source Data Fig. 4 Statistical source data.

Source Data Fig. 5 Statistical source data.

Source Data Fig. 6 Statistical source data.

Source Data Extended Data Fig. 1 Statistical source data.

Source Data Extended Data Fig. 2 Statistical source data.

Source Data Extended Data Fig. 3 Statistical source data.

Source Data Extended Data Fig. 4 Statistical source data.

Source Data Extended Data Fig. 6 Statistical source data.

Source Data Extended Data Fig. 7 Statistical source data.

## Data Availability

Raw nCounter data that support the findings of this study have been deposited in the NCBI GEO under accession no. GSE232529. Data used for the melanoma CPI response analyses were downloaded as supplementary tables from Liu et al.^33^ and from the GEO under the following accession numbers: GSE115821 (ref. ^32^), GSE168204 (ref. ^34^), GSE78220 (ref. ^30^) and GSE91061 (ref. ^31^). Source data are provided with this paper. All other data needed to understand and evaluate the conclusions of the paper are provided in the manuscript and supplementary material.

## Code availability

No custom code was generated for this study.
