# Supplementary material for: PD-1 defines a distinct, functional, tissue-adapted state in Vδ1+ T cells with implications for cancer immunotherapy
Source: Nat Cancer. 2024 Jan 3;5(3):420–32. doi: 10.1038/s43018-023-00690-0 (PMC10965442; doi:10.1038/s43018-023-00690-0)
Supplement: Supplementary file 1 — Reporting Summary [file 43018_2023_690_MOESM1_ESM.pdf]

# nature portfolio

Corresponding author(s): Adrian Hayday
Yin Wu

Last updated by author(s): Sep 26, 2023

# Reporting Summary

Nature Portfolio wishes to improve the reproducibility of the work that we publish. This form provides structure for consistency and transparency in reporting. For further information on Nature Portfolio policies, see our Editorial Policies and the Editorial Policy Checklist.

## Statistics

For all statistical analyses, confirm that the following items are present in the figure legend, table legend, main text, or Methods section.

| n/a | Confirmed | |
|---|---|---|
| ☐ | ☒ | The exact sample size (*n*) for each experimental group/condition, given as a discrete number and unit of measurement |
| ☐ | ☒ | A statement on whether measurements were taken from distinct samples or whether the same sample was measured repeatedly |
| ☐ | ☒ | The statistical test(s) used AND whether they are one- or two-sided<br>*Only common tests should be described solely by name; describe more complex techniques in the Methods section.* |
| ☒ | ☐ | A description of all covariates tested |
| ☐ | ☒ | A description of any assumptions or corrections, such as tests of normality and adjustment for multiple comparisons |
| ☐ | ☒ | A full description of the statistical parameters including central tendency (e.g. means) or other basic estimates (e.g. regression coefficient) AND variation (e.g. standard deviation) or associated estimates of uncertainty (e.g. confidence intervals) |
| ☐ | ☒ | For null hypothesis testing, the test statistic (e.g. *F*, *t*, *r*) with confidence intervals, effect sizes, degrees of freedom and *P* value noted<br>*Give P values as exact values whenever suitable.* |
| ☒ | ☐ | For Bayesian analysis, information on the choice of priors and Markov chain Monte Carlo settings |
| ☒ | ☐ | For hierarchical and complex designs, identification of the appropriate level for tests and full reporting of outcomes |
| ☒ | ☐ | Estimates of effect sizes (e.g. Cohen's *d*, Pearson's *r*), indicating how they were calculated |

*Our web collection on statistics for biologists contains articles on many of the points above.*

## Software and code

Policy information about availability of computer code

| | |
|---|---|
| Data collection | No custom code was used to collect data.<br>BD FACSDiva software version 9.0 was used for collection of flow cytometry data.<br>A NanoString nCounter digital analyzer (Max) was used for collection of transcriptomic data. |
| Data analysis | No custom code was used for data analysis.<br><br>FlowJo version 10<br>GraphPad Prism version 9.5.0<br>JMP Pro version 17.0.0 |

For manuscripts utilizing custom algorithms or software that are central to the research but not yet described in published literature, software must be made available to editors and reviewers. We strongly encourage code deposition in a community repository (e.g. GitHub). See the Nature Portfolio guidelines for submitting code & software for further information.

# Data

Policy information about availability of data

All manuscripts must include a data availability statement. This statement should provide the following information, where applicable:

- Accession codes, unique identifiers, or web links for publicly available datasets
- A description of any restrictions on data availability
- For clinical datasets or third party data, please ensure that the statement adheres to our policy

Raw nCounter data that support the findings of this study have been deposited in the NCBI Gene Expression Omnibus (GEO) under accession no. GSE232529. Data used for melanoma CPI response analyses were downloaded as supplemental tables from Liu et al. (PMID: 31792460) and from the Gene Expression Omnibus under the following accession numbers: (GSE115821), (GSE168204), (GSE78220) and (GSE91061). All other necessary data to understand and evaluate the conclusions of the paper are provided in the manuscript and supplemental material. Data are available from the corresponding authors upon reasonable request.

# Research involving human participants, their data, or biological material

Policy information about studies with human participants or human data. See also policy information about sex, gender (identity/presentation), and sexual orientation and race, ethnicity and racism.

| | |
|---|---|
| Reporting on sex and gender | Sex/gender were not considered in study design. The study includes both male and female skin donors. All NSCLC donors were male. Sex was determined based on assigned biological sex. |
| Reporting on race, ethnicity, or other socially relevant groupings | Data on race, ethnicity or other socially relevant groupings were not routinely collected as part of either study and were not controlled for. |
| Population characteristics | Skin donors were a subset of participants recruited to the study: Immune surveillance in the skin: Implications of unconventional lymphocytes in health, inflammation and cancer.<br><br>Human NSCLC samples were supplied by the King's Health Partners Cancer Biobank. A summary of patient demographics is provided in Extended Data Table 1. |
| Recruitment | Skin samples that would otherwise have been discarded were obtained from participants undergoing elective plastic and reconstructive surgical procedures. Anatomically, samples were either derived from breast or abdomen. All donors gave written informed consent.<br><br>Human NSCLC samples and clinical data were supplied by the King's Health Partners Cancer Biobank. All patients provided written informed consent for collection of tissues excess to diagnostic requirements by the King's Health Partners Cancer Biobank. |
| Ethics oversight | Title: Immune surveillance in the skin: Implications of unconventional lymphocytes in health, inflammation and cancer.<br>REC: 15/LO/2130<br>IRAS: 169471<br>The study was approved by the London - Camberwell and St Giles Research Ethics Committee.<br><br>Title: King's Health Partners Cancer Biobank<br>REC: 18/EE/0025<br>IRAS: 240747<br>The study was approved by the East of England - Cambridge East Research Ethics Committee. |

Note that full information on the approval of the study protocol must also be provided in the manuscript.

# Field-specific reporting

Please select the one below that is the best fit for your research. If you are not sure, read the appropriate sections before making your selection.

☒ Life sciences ☐ Behavioural & social sciences ☐ Ecological, evolutionary & environmental sciences

For a reference copy of the document with all sections, see nature.com/documents/nr-reporting-summary-flat.pdf

# Life sciences study design

All studies must disclose on these points even when the disclosure is negative.

| | |
|---|---|
| Sample size | The sample size was not predetermined. Samples were chosen based on the availability of banked materials. No other selection criteria were used. |

| | |
|---|---|
| Data exclusions | No data were excluded from analyses. |
| Replication | In vitro activation assays were undertaken to validate transcriptomic analyses. Owing to the limited nature of clinical samples, technical replicates were not performed. All statistics were derived from biological replicates. |
| Randomization | Non-interventional study, not applicable |
| Blinding | Non-interventional study, not applicable |

# Reporting for specific materials, systems and methods

We require information from authors about some types of materials, experimental systems and methods used in many studies. Here, indicate whether each material, system or method listed is relevant to your study. If you are not sure if a list item applies to your research, read the appropriate section before selecting a response.

## Materials & experimental systems

| n/a | Involved in the study | |
|---|---|---|
| ☐ | ☒ | Antibodies |
| ☒ | ☐ | Eukaryotic cell lines |
| ☒ | ☐ | Palaeontology and archaeology |
| ☒ | ☐ | Animals and other organisms |
| ☒ | ☐ | Clinical data |
| ☒ | ☐ | Dual use research of concern |
| ☒ | ☐ | Plants |

## Methods

| n/a | Involved in the study | |
|---|---|---|
| ☒ | ☐ | ChIP-seq |
| ☐ | ☒ | Flow cytometry |
| ☒ | ☐ | MRI-based neuroimaging |

## Antibodies

Antibodies used

Antibody -> Supplier -> Catalog Number -> Clone -> Dilution used
Anti-CD107a – BV650 -> Biolegend -> 328638 -> H4A3 -> 1:400
Anti-CD14 – APC-Cy7 -> Biolegend -> 301820 -> M5E2 -> 1:100
Anti-CD19 – APC-Cy7 -> Biolegend -> 302218 -> HIB19 -> 1:100
Anti-CD45 – Pacific Blue -> Biolegend -> 304029 -> HI30 -> 1:100
Anti-CD68 – APC-Cy7 -> Biolegend -> 333822 -> Y1(/82A) -> 1:100
Anti-CD8a – AF700 -> Biolegend -> 300920 -> HIT8a -> 1:100
Anti-CD8a – FITC -> BD Biosciences -> 345772 -> SK1 -> 1:100
Anti-IFNg – PE -> Biolegend -> 502509 -> 4S.B3 -> 1:100
Anti-IL-17A – BV605 -> Biolegend -> 512325 -> BL168 -> 1:100
Anti-PD-1 – BV421 -> Biolegend -> 329920 -> EH12.2H7 -> 1:100
Anti-TCR-Vd1 - FITC -> Miltenyi -> 130-118-362 -> REA173 -> 1:200
Anti-TCR-Vd1 – APC -> Miltenyi -> 130-118-968 -> REA173 -> 1:200
Anti-TCR-Vd1 - FITC -> Thermo Fisher -> TCR2730 -> TS8.2 -> 1:100
Anti-TCRab – PE -> Biolegend -> 306708 -> IP26 -> 1:100
Anti-TCRab – PerCP-Cy5.5 -> Biolegend -> 306724 -> IP26 -> 1:100
Anti-TCRgd – PE-Cy7 -> Beckman Coulter -> B10247 -> IMMU510 -> 1:100
Anti-TNFa – APC -> Biolegend -> 502912 -> MAb11 -> 1:100
Anti-TCR- Vd2 – APC-Cy7 -> Biolegend -> 331440 -> B6 -> 1:100
Anti-Ki67 BV421 -> Biolegend -> 350506 -> Ki-67 -> 1:100
Live/Dead Zombie– NIR -> Biolegend -> 423105 -> NA -> 1:1000
TCR Vd1 antibody -> Miltenyi -> 130-122-285 -> REA173 -> 10ug/ml
Ultra-LEAF Purified mouse IgG2a -> Biolegend -> 400264 -> MOPC-173 -> 30ug/ml
LEAF Purified anti-human CD3 -> Biolegend -> 317304 -> OKT3 -> 1ug/ml
Recombinant human PD-L1 Fc chimera -> Biolegend -> 762506 -> NA -> 10ug/ml
Recombinant human MICA Fc chimera protein CF -> Bio-techne -> 1300-MA -> NA -> 10ug/ml

Validation

Antibodies and reagents used have been validated by commercial suppliers and validation statements can be found on supplier websites.

BD Biosciences
https://www.bdbiosciences.com/en-gb/products/reagents/flow-cytometry-reagents/research-reagents/quality-and-reproducibility
"Antibody specificity. BD Biosciences not only develops its own antibodies but also collaborates with research scientists around the world to license their antibodies. We provide accessibility to the flow cytometry community by conjugating antibodies to a broad portfolio of high-performing dyes, including our vastly popular portfolio of BD Horizon Brilliant™ Dyes. A world-class team of research scientists helps ensure that these reagents work reliably and consistently for flow cytometry applications. The specificity is confirmed by using multiple applications that may include a combination of flow cytometry, immunofluorescence, immunohistochemistry or western blot to test a combination of primary cells, cell lines or transfectant models.All flow cytometry reagents are titrated on the relevant positive or negative cells. To save time and cell samples for researchers, pre-titrated test size reagents are bottled at an optimal concentration, with the best signal-to-noise ratio on relevant models. You can look up the Certificate of Analysis and the concentration of test-size human reagents from specific lots via the Concentration Lookup page or BD Regulatory Documents.

Technical data sheets provide data generated on the relevant primary model at this optimal concentration based on a titration curve. QC data on any lot of reagent can be requested through ResearchApplications@bd.com."

Beckman Coulter
https://www.beckman.com/reagents/coulter-flow-cytometry
"Our portfolio of Flow Cytometry reagents, entirely manufactured under good manufacturing practices (GMP), covers major application areas including hemato-oncology, HIV analysis, immune monitoring, cell cycle and stem cells studies."

BioLegend
https://www.biolegend.com/en-us/quality/quality-control
"All of our products undergo industry-leading rigorous quality control (QC) testing to ensure the highest level of performance and reproducible results. Each lot is compared to an internally established "gold standard" to maintain lot-to-lot consistency. We also conduct wide-scale stability studies to guarantee an accurate shelf-life for our products.Additionally, we test the majority of our products on endogenous cells rather than transfected or immortal cells that may overexpress the analyte. We assess our reagents with samples and protocols that reflect our customers' experience. Our willingness to monitor the quality of our reagents extends beyond our lab and into yours. Flow Cytometry Reagents. Specificity testing of 1-3 target cell types with either single- or multi-color analysis (including positive and negative cell types). Once specificity is confirmed, each new lot must perform with similar intensity to the in-date reference lot. Brightness (MFI) is evaluated from both positive and negative populations. Each lot product is validated by QC testing with a series of titration dilutions."

Bio-techne
https://www.bio-techne.com/reagents/proteins
"High Levels of Biological Activity: The biological activity of each protein is tested in an appropriate biological system to confirm that it meets our strict QC activity parameters before it is released to market. Lot-to-Lot Consistency: Minimal lot-to-lot variability is ensured by maintaining consistent manufacturing conditions and testing each new lot side-by-side with previous lots, so you don't have to worry whether results will be reproducible over time. High Purity: The majority of our proteins have >95% purity and a guaranteed industry-leading endotoxin level of <0.1 EU/ug by the LAL method. Long-term Stability: The bioactivity and physical state of our proteins are monitored through multiple freeze/thaw cycles, so you can be confident that your protein will be stable over long periods of time. All proteins are shipped lyophilized or in specially optimized formulations to ensure that they reach you in perfect condition. Comprehensive Selection: Our catalog includes over 5,000 biologically active recombinant and natural proteins spanning 35 species, and more than 10,000 non-catalog proteins for multiple different research areas, allowing you to easily find the proteins that you need. Multiple Host Expression Systems, Tags, and Labels: Our proteins are produced in a variety of host expression systems and are either untagged or include a biotin, Fc, or His tag, or a fluorescent label to meet different experimental needs. Seamless Transition from RUO to GMP: Our Animal-free and GMP-grade proteins frequently originate from the same clone, sequence, and expression system as our traditional research-grade proteins, making the transition from basic research into process development and clinical manufacturing as efficient and seamless as possible."

Miltenyi
https://www.miltenyibiotec.com/GB-en/products/macs-antibodies/antibody-validation.html
"Antibody validation. Standardized antibody validation methods. High purity, lot-to-lot consistency, and antibody reproducibility. Reliable antibody specificity and sensitivity. All our antibodies are rigorously tested and validated before release. In the application section on the product page, you can find examples of typical performance data. In addition, we provide extended validation data highlighting details of antibody performance, specificity, and fixation compatibility."

ThermoFisher
https://www.thermofisher.com/uk/en/home/life-science/antibodies/invitrogen-antibody-validation.html
"Invitrogen antibodies are currently undergoing a rigorous two-part testing approach.

Part 1 - Target specificity verification. This helps ensure the antibody will bind to the correct target. Our antibodies are being tested using at least one of the following methods to ensure proper functionality in researcher's experiments. Click on each testing method below for detailed testing strategies, workflow examples, and data figure legends.
• Knockout—expression testing using CRISPR-Cas9 cell models
• Knockdown—expression testing using RNAi to knockdown gene of interest
• Independent antibody verification (IAV)—measurement of target expression is performed using two differentially raised antibodies recognizing the same protein target
• Cell treatment—detecting downstream events following cell treatment
• Relative expression—using naturally occurring variable expression to confirm specificity
• Neutralization—functional blocking of protein activity by antibody binding
• Peptide array—using arrays to test reactivity against known protein modifications
• SNAP-ChIP™—using SNAP-ChIP to test reactivity against known protein modifications
• Immunoprecipitation-Mass Spectrometry (IP-MS)—testing using immunoprecipitation followed by mass spectrometry to identify antibody targets

Part 2 - Functional application validation
These tests help ensure the antibody works in a particular application(s) of interest, which may include (but are not limited to):
• Western blotting
• Flow cytometry
• ChIP
• Immunofluorescence imaging
• Immunohistochemistry
Most antibodies were developed with specific applications in mind. Testing that an antibody generates acceptable results in a specific application is the second part of confirming antibody performance."

# Flow Cytometry

## Plots

Confirm that:

☒ The axis labels state the marker and fluorochrome used (e.g. CD4-FITC).

☒ The axis scales are clearly visible. Include numbers along axes only for bottom left plot of group (a 'group' is an analysis of identical markers).

☒ All plots are contour plots with outliers or pseudocolor plots.

☒ A numerical value for number of cells or percentage (with statistics) is provided.

## Methodology

| | |
|---|---|
| Sample preparation | Cells were washed twice in PBS to remove traces of serum before staining with Zombie NIR viability dye at 1:1000 dilution in PBS for 15 minutes at room temperature. Cells for downstream NanoString transcriptomics were stained for 20 minutes at 4oC. Cells were then stained with an antibody cocktail (see antibody table below) against surface markers for 20 minutes at 4°C in FACS buffer, washed twice and then resuspended in FACS buffer (PBS + 1 mM EDTA + 2% FCS v/v) prior to acquisition on a BD LSRFortessa or sorting on a BD FACSAria Fusion. For intracellular cytokine staining, samples were fixed after surface staining using BD CellFIX followed by two washes with Perm/Wash Buffer (BioLegend) and intracellular staining with an antibody cocktail for 30 minutes at 4oC in perm/wash buffer (see antibody table below). After 30 minutes, samples were washed twice with Perm/Wash buffer and resuspended in FACS buffer prior to acquisition on a BD LSRFortessa. FCS3.0 files were analysed using FlowJo v.10. |
| Instrument | BD LSRFortessa or BD FACSAria Fusion (cell sorting). |
| Software | FCS files were analysed using FlowJo version 10. |
| Cell population abundance | An average of 13,650 ± 2,355 cells (mean ± S.D.) were sorted for each cell population analysed by NanoString. |
| Gating strategy | Preliminary gates selected cells using an FSC and SSC gate. Doublets were excluded by plotting FSC area versus FSC height and excluding cells outside of a linear correlation. Dead cells were excluded by gating for Live-Dead Zombie - NIR low cells. Subsequent gating was as described in supplementary data panels. |

☒ Tick this box to confirm that a figure exemplifying the gating strategy is provided in the Supplementary Information.
